# The Weak Shall Inherit: Bacteriocin-Mediated Interactions in Bacterial Populations

**DOI:** 10.1371/journal.pone.0063837

**Published:** 2013-05-21

**Authors:** Hadeel Majeed, Adam Lampert, Lusine Ghazaryan, Osnat Gillor

**Affiliations:** 1 Zuckerberg Institute for Water Research, J. Blaustein Institutes for Desert Research, Ben-Gurion University, Sede Boqer Campus, Midreshet Ben-Gurion, Israel; 2 Department of Physics of Complex Systems, Weizmann Institute of Science, Rehovot, Israel; The Scripps Research Institute, United States of America

## Abstract

**Background:**

Evolutionary arms race plays a major role in shaping biological diversity. In microbial systems, competition often involves chemical warfare and the production of bacteriocins, narrow-spectrum toxins aimed at killing closely related strains by forming pores in their target’s membrane or by degrading the target’s RNA or DNA. Although many empirical and theoretical studies describe competitive exclusion of bacteriocin-sensitive strains by producers of bacteriocins, the dynamics among producers are largely unknown.

**Methodology/Principal findings:**

We used a reporter-gene assay to show that the bacterial response to bacteriocins’ treatment mirrors the inflicted damage Potent bacteriocins are lethal to competing strains, but at sublethal doses can serve as strong inducing agents, enhancing their antagonists’ bacteriocin production. In contrast, weaker bacteriocins are less toxic to their competitors and trigger mild bacteriocin expression. We used empirical and numerical models to explore the role of cross-induction in the arms race between bacteriocin-producing strains. We found that in well-mixed, unstructured environments where interactions are global, producers of weak bacteriocins are selectively advantageous and outcompete producers of potent bacteriocins. However, in spatially structured environments, where interactions are local, each producer occupies its own territory, and competition takes place only in “no man’s lands” between territories, resulting in much slower dynamics.

**Conclusion/Significance:**

The models we present imply that producers of potent bacteriocins that trigger a strong response in neighboring bacteriocinogenic strains are doomed, while producers of weak bacteriocins that trigger a mild response in bacteriocinogenic strains flourish. This counter-intuitive outcome might explain the preponderance of weak bacteriocin producers in nature. However, the described scenario is prolonged in spatially structured environments thus promoting coexistence, allowing migration and evolution, and maintaining bacterial diversity.

## Introduction

Species that comprise highly diverse communities are often engaged in a fierce arms race over resources and space. To overcome their adversaries, species, large and small, use every weapon in their arsenal including secondary metabolites, extracellular enzymes, or antibiotics, [1], [2]. Competitions between bacteria, Archaea, Fungi or Protozoa are often resolved by the use of antimicrobials. The leading antibiotics used in the bacteria and archaea world are bacteriocins [3] – proteinaceous toxins that enable their producing organisms to defend their habitat against invaders, limit the advance of neighboring cells [4] or invade an established bacterial community [5], [6].

A model system for investigating the mechanisms of bacteriocin structure, function, ecology and evolution are the colicins, named after their producing species *Escherichia coli*. Colicins are high-molecular-weight toxic proteins that kill closely related species through a variety of mechanisms. Most of the characterized colicins (and bacteriocins) make pores in their antagonists’ inner membrane, while the others degrade either the DNA or RNA of the target cell [7]. Colicin-producing populations are safe from harm as the colicin-encoding gene is tightly linked to a gene conferring immunity to the toxin [8]. Moreover, due to the lack of a colicin-secreting system that would transfer the toxin from the producing cell’s cytoplasm to the environment, emission of colicin requires the colicinogenic cell to die by lysis [7]. Accordingly, colicin production is harmful to both the producing cell and its target. However, under natural conditions, only a small proportion of the population (>3%) will go through the production of colicin and consequent lysis [9].

Theoretical and empirical studies have reported conditions that favor the maintenance of the costly trait of bacteriocin production in both population and community settings [4], [10], [11], [12], [13]. In an unstructured environment with global interactions among free-swimming cells, a small population of producers was unable to invade an established population of sensitive cells [12], [15] due to the high cost and low gain. The producers pay dearly for the toxins they secrete as they lyse during the process. Yet, in well-mixed environments, the benefits (i.e., the resources made available by killing sensitive organisms) are randomly distributed to be enjoyed indiscriminately by all cells. Therefore, colicin producers were shown to prevail over the non-producers only when they are above a certain threshold [11], [14].

In a spatially structured environment with local interactions among sessile colonies, the benefit to the bacteriocin-producing colony is more immediate, and thus colicinogenic strains can increase in frequency, even when initially rare and displace sensitive strains [12], [15]. When an additional player is added, one that is resistant to colicin, local diversity is promoted as the trio engages in a rock-paper-scissors (RPS) type game in which the producers toxify the sensitive, the resistant blunts the producer (due to the costs owed to the production of colicins) but the sensitive defeats the resistant (due to the cost of resistance). This trio’s dynamics resolves in favor of the resistant strain in unstructured and semi-structured environments, but in a structured environment, the three strains coexist [4], [14].

The RPS model accurately predict colicin-mediated interactions, yet, recently published observations demonstrated that natural colicinogenic populations are limited to producer and resistant strains, almost completely excluding sensitive strains. *E. coli* strains were isolated from mammalian gut samples, and 10 to 50% were found to produce at least one colicin, while resistant strains were abundant, ranging from 50 to 90% of the population [16], [17], [18], [19]. In comparison, the sensitive population was very small (less then 5%). These observations are puzzling; if the interactions among colicinogenic strain are not ruled by the RPS model, what are the key dynamics favoring producers coexistence?

To address this question, we explored the interactions between two colicinogenic strains, both kill by degrading the DNA of their target cells [19]. We found that these strains mutually induce the expression of each other’s bacteriocin. Competitions assays further showed that in an unstructured environment the slightly more toxic of the two strains displaced its less potent opponent. Localized interactions resulted in the two strains coexisting in a spatially “frozen” pattern, suggesting that mutual induction leads to mutual exclusion and coexistence [19]. Following these results we wondered whether the proposed model applies to all bacteriocin-mediated interactions, regardless of the bacteriocin’s mode of action.

Here, we explored the expression of colicin in colicinogenic strains induced by challenging colicins with different modes of action. We hypothesized that potent colicins would trigger strong expression in colicinogenic strains, while induction by weaker colicins would result in milder response. We further hypothesized that pairwise competition between strains engaged in cross-induction will be resolved by the strains producing potent and strong triggering colicins prevailing over weaker adversaries. We predicted that strong inducers would dominate the community in unstructured environments, but that in structured environments, both strains would coexist for prolonged periods due to local interactions between colonies.

## Materials and Methods

### Bacterial Strains and Plasmids

The bacteriocin plasmids used in this study are listed in Table 1. Each plasmid was extracted from its original host using the AccuPrep Kit (BioNeer, Seoul, South Korea) and transformed into *E. coli* strain BZB1011 [20], providing an isogenic background for the different bacteriocin-encoding plasmids. Transformants were selected on the basis of bacteriocin production and their identities were confirmed by PCR (data not shown). The growth rate of each of the strains used in this study was evaluated (Table S1) and found to be similar for all strains.

**Table 1:** Bacterial strains and plasmids used in this study.

Strain	Identification	Description	Ref
**Bacteria**			
BZB1011	BZB1011	W3110; *gyrA*	[21]
**Plasmids** *			
pDEW201	DEW201	promoterless plx, *luxCDABE*(-)Amp^r^	[35,38]
pDEW-A	Lum-A	pDEW201(P*caa*::*luxCDABE*) Amp^r^	*This study*
pDEW-B	Lum-B	pDEW201(P*cba*::*luxCDABE*) Amp^r^	*This study*
pDEW-D	Lum-D	pDEW201(P*cda*::*luxCDABE*) Amp^r^	*This study*
pDEW-E2	Lum-E2	pDEW201(P*ce2a*::*luxCDABE*) Amp^r^	[21]
pDEW-E6	Lum-E6	pDEW201(P*ce6a*::*luxCDABE*) Amp^r^	*This study*
pDEW-E7	Lum-E7	pDEW201(P*ce7a*::*luxCDABE*) Amp^r^	[22]
pDEW-Ib	Lum-Ib	pDEW201(P*ciba*::*luxCDABE*) Amp^r^	[22]
pDEW-K	Lum-K	pDEW201(P*cka*::*luxCDABE*) Amp^r^	*This study*
pCol A-CA31	ColA	Colicin A plasmid	[21]
pCol D-CA23	ColD	Colicin D plasmid	[21]
pColE2-P9	ColE2	Colicin E2 plasmid	[21]
pCol E6-CT14	ColE6	Colicin E6 plasmid	[21]
pColE7-K317	ColE7	Colicin E7 plasmid	[21]
pColIb-P9 drd	ColIb	Colicin Ib plasmid	[21]
pCol K-K253	ColK	Colicin K plasmid	[21]
pUA*rrnB*	pUA*rrnB*	pUA P*rrnB*::*gfp*, Kan^R^	[36]
ColA-pBR322	pBR-ColA	pBR *caa*::*ima*::*cal*, Amp^R^	*This study*

* p indicates plasmid, all plasmids were transformed into isogenic host BZB1011.

### Plasmid Construction

The promoter regions of colicins A, D, E6 and K were amplified (the primers used are listed in Table S2) as previously described [22] and the amplicons were fused upstream of *Photorhabdus luminescence lux*CDABE reporter [23], [24]. The resulting reporter vectors were transformed into the colicin-sensitive *E. coli* strain BZB1011 (Table 1). Colicinogenic plasmid: Colicin A operon [including the genes encoding the toxin (accession number X01008), immunity (accession number X00964) and lysis (accession number X02391)] was PCR-amplified (the primers used are listed in Table S2). The resulting amplicon and pBR322 vector were digested with *Eco*RI and *Hind*III restriction enzymes (Fermentas, Vilnius, Lithuania) and fused to form the pBR322-ColA plasmid. The plasmid was transformed into *E. coli* strain BZB1011 together with the constitutively fluorescent plasmid pUA*rrnB* [25]. The resulting strain ColA-pUA*rrnB* was used for the competition assays described below. To find out whether the constructed strain is equal in potency and fitness to the native ColA strain (Table 1), we have competed the two strains as described below. This competition was not resolved; both strains were detected at equal frequencies after 24 hours (data not shown) suggesting that the constructed strain is equivalent to the naïve strain.

### Growth Conditions

M9 minimal salt (Sigma, St. Louis, MO) and Luria-Bertani broth and agar (Difco, Lawrence, KS) were prepared according to the manufacturers’ instructions. The media were supplemented with 100 µg mL^−1^ ampicillin and 50 µg mL^−1^ kanamycin as required. Cultures were grown in an incubator (New Brunswick, Edison, NJ) at 37°C with shaking at 200 rpm.

### Colicin Titer Assay

Colicin extracts were prepared as previously described [26], [18] and stored at –80 °C until use. Colicin titer assays were used to measure the potency of each colicin. The assays were performed as previously described [5] with minor alterations. Briefly, all colicin extracts were serially diluted and 20 µL of each dilution was spotted on a lawn of the sensitive strain (*E. coli* strain BZB1011). Colicinogenic strains ColA, ColD, ColK, ColIb, ColE2, ColE6 and ColE7, in addition to the colicin-free isogenic host strain (Table 1), were grown to mid log phase and then induced by adding 0.5 ng µL^−1^ mitomycin C (Sigma), which is a known inducer of colicin production [7]. We demonstrated that induction of colicin promoter at this mitomycin C concentration triggers similar colicin expression, with the exception of colicins A and Ib (Table S3). The colicin extracts were serially diluted and 20 µL of each dilution were spotted onto LB solid agar plate inoculated with a lawn of colicin-sensitive indicator *E. coli* strain BZB1011. The titer of the colicin was taken as the inverse of the greatest dilution that still results in a clear inhibition zone in the indicator.

### Reporter Assay

To determine the optimal concentration of colicin extract for induction of colicin production, each colicin extract was tested on the reporter strain controlled by the promoter of colicin E2 (E2-Lum; Table 1). Using a series of double dilutions (PBS; Sigma), 28 concentrations of each colicin were screened to identify those resulting in optimum bioluminescence. The experiment was conducted as previously described [22]. The reporter strains were grown in LB broth supplemented with ampicillin. The cultures were diluted in LB (1∶100, v/v), grown to early log phase (OD_600_ = 0.09) and treated with various concentrations of colicin extracts. Duplicates of each concentration were incubated together with the reporter strain in 96-well microtiter plates (Grainer, Frickenhausen, Germany) and monitored for light emission (λ = 600 nm) in a temperature-controlled plate reader (Infinite M200, Tecan, Grödig, Austria) for 5 h at hourly intervals. Reporter assays were performed in accordance with the preliminary experiments, using the seven double dilutions ranging from lethal to sub-lethal concentrations of the colicin extract (chosen according to the preliminary experiments as described above). Plates holding the seven dilutions of each colicin and all the reporter strains (Table 1) were incubated in the plate reader (Infinite M200) and the emitted light was monitored for 5 h at 1–h intervals. All experiments were run in duplicate and repeated at least three times. The results are reported in arbitrary relative light units (RLU). Luminescence values are presented as the ratio of the induced reporters’ luminescence to the non-induced control (response ratio) as previously described [22].

### Competition Experiments in an Unstructured Environment

Following the reporter assays (Table 2), we tested a subsample of the strains for their competitive advantage. We used three colicinogenic strain types: (i) a potent toxin and strong inducer, ColE7; (ii) an intermediate toxin and intermediate inducer, ColD, and (iii) mild toxins provoking mild induction, ColA, D and E6.

**Table 2:** Monitoring colicin expression using reporter vectors triggered by colicin extracts.

Function	*DNA degradation*		*RNA degradation*		*Pore-formation*					*Control*	
Colicin	*ColE2*	*ColE7*	*ColE6*	*ColD*		*ColA*		*ColK*	*ColIb*		*BZB*
Dilution	3×10^−6^	3×10^−3^	3×10^−5^	3×10^−2^	3×10^−2^		8×10^−4^		4×10^−1^		1×10^0^
*Lum-E2*	**126.9±5.9**	**141.8±2.7**	1.2±.05	**42.0±4.4**	1.5±0.1		**5.2±0.5**		**9.4±0.8**		1.1±0.10
*Lum-E7*	**135.9±9.2**	**140.5±2.1**	1.3±0.2	**55.5±8.9**	1.5±0.2		**2.8±0.3**		**15±3.2**		1.0±0.07
*Lum-E6*	**67.8±2.2**	**94.3±4.3**	1.1±0.1	**36.6±0.4**	1.3±0.1		**3.4±0.6**		**11±0.3**		1.0±0.05
*Lum-D*	**107.8±5.4**	**117.5±2.0**	1.0±0.05	**48.0±6.0**	2.1±0.3		**2.1±0.5**		**9.2±0.4**		1.0±.050
*Lum-A*	**91.9±7.4**	**103.0±4.4**	1.4±0.35	**56.6±4.1**	1.6±0.2		**2.2±0.3**		**13±1.3**		1.0±0.02
*Lum-K*	**88.2±4.5**	**109.7±5.2**	1.4±0.5	**44.0±2.5**	1.6±0.1		**3.3±0.5**		**9.0±2.4**		1.0±0.03
*Lum-Ib*	**44.5±7.0**	**42.4±9.0**	3.5±0.25	**11.7±1.8**	1.1±0.2		1.7±0.7		**2.3±0.1**		0.9±0.30
*Lum-P(–)*	1.8±0. 5	1.6±0.7	0.9±0.04	1.8±0.1	1.1±0.1		1.6±.04		1.3±0.15		1.0±0.00

Data presented as mean ± standard deviation. Threshold set for induction: RLU/RLU_0_>2.

To explore the outcome of competition in an unstructured environment, when interactions between populations are global, the reporter strain ColA-pUA*rrnB* (Table 1) was set to compete against the five strains mentioned above. The pairwise competitions were performed at varying initial frequencies of the competing strains: 50%, 10%, 2%, 0.4%, 0.08%, 0.016%, 0.003% and 0%. As a control, we used the colicin-free isogenic host strain. The colicinogenic strains’ competitions were monitored in real time (Infinite M200) by measuring the fluorescence emitted by the reporter strain such that only the tagged competitor was detected. Moreover, the reporter gene used in this study carried *gfpmut2* [27]: this gene encodes a protein that is non-degradable such that cell death does not diminish fluorescent levels [25]. Therefore, a halt in fluorescence accumulation translates to cessation in the reporter’s growth. The results should be interpreted by inferring the competitiveness of each of the reporter’s opponents from the reporter’s status.

### Competition Experiments in a Structured Environment

The plate competition assays were performed as previously described [4], [19]. Briefly, the pairwise competitions employed the isogenic host strain harboring the ColA-pBR322 plasmid against strains ColE7, E6, and K (Table 1). Each day, the plates depicting the competition were photographed and the photos were analyzed for percentage of areal coverage of each strain using NIH IMAGE E software (http://rsb.info.nih.gov/ij/index.html). Each experiment was performed in duplicate with different randomized lattice inoculation.

### Simulations

We simulated both a homogeneous system allowing global interaction corresponding to an unstructured environment, and a spatial system allowing local interaction corresponding to a structured environment. To that end, the promoter regions of the genes encoding colicins A, D, E2, E6, E7, Ib, and K were fused upstream of the *Photorhabdus luminescence luxCDABE* reporter operon (Table 1) and transformed into *E.* Our theoretical examination was based on the analysis of four adjacent continuous variables (Table 3), namely the frequency of the test bacteria, strain A (*u_A_*), the frequency of the competitor bacteria, strain B (*u_B_*), and the potency of their respective bacteriocins, *c_A_* and *c_B_*. We assumed that both bacteria have the same growth-rate, β, death rate, δ, and carrying capacity normalized to 1. The bacteria differed in their sensitivity γ^i^ (*i* = 1, 2) and response *l_i_* (induction rate) to the competitor bacterial bacteriocin. The dynamics of the homogeneous system is thus given by the following differential equations:
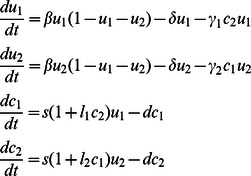
where *s* is bacteriocin production without induction, and *d* is the disassembly rate of the bacteriocins. In spatial systems, variables depend on both space and time. In addition, we assume spreading of bacteria on the surface and diffusion of colicins, and therefore the terms 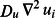 and 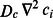 are added to the equations. Simulations were performed in 1D and 2D.

**Table 3:** Description of simulation parameters and variables.

Symbol	Notation
*u_i_*	Frequency of strain *i* (*i* = 1;2)
*c_i_*	Density of bacteriocin (produced by species *i*)
*β_i_*	Growth rate of strain *i*
*δ*	Death rate of strain that is not exposed to bacteriocins
*γ_i_*	Toxicity of bacteriocin *i*
*s*	Bacteriocin production rate without induction
*l_i_*	Induction rate of bacteriocin *i* of strain *i* in response to a competitor’s bacteriocin
*d*	Disassembly rate of the bacteriocins

## Results

### Colicin-colicin Potency and Induction

**Figure 1:**
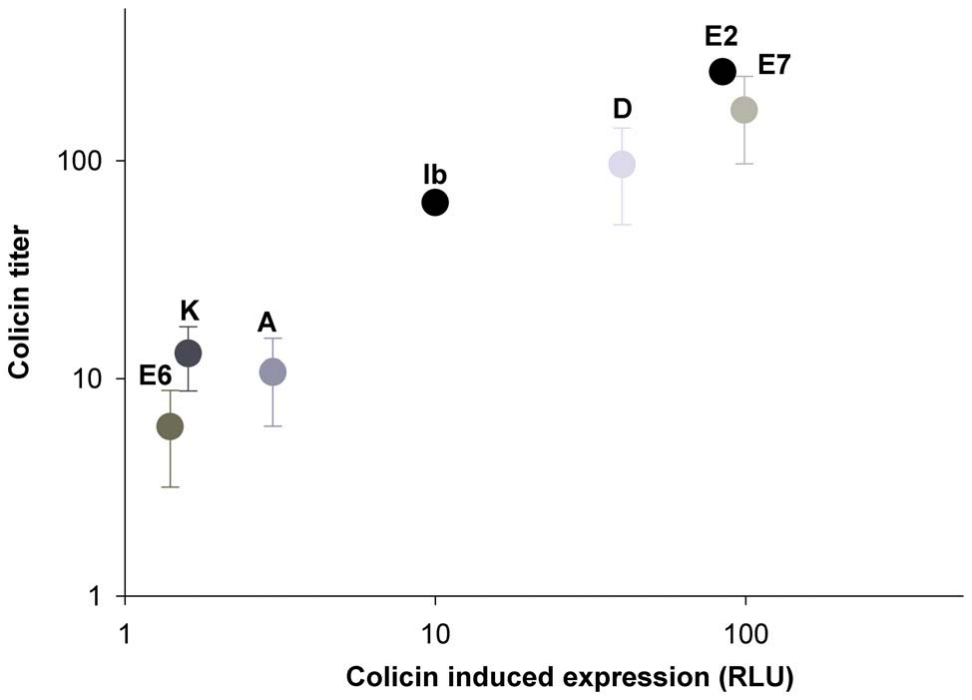
Colicin toxicity and cross-induction. The relationship between colicins titer and the expression these colicin extracts triggered in the reporter strains (Table 1) were tested. The colicins’ titer correlated with the measured light emitted by the reporter strains. Results are presented as average with standard deviation of the light emission of all reporters tested. Each experiment was preformed in duplicates and repeated at least three times.

We began by examining colicins’ potency using the spot titer assay [18]. All colicin producing strains and their isogenic host lacking the colicin-encoding vector (Table 1) were induced to produce colicins, but not before we demonstrated that the treatment we applied prompt similar expression in the different colicinogenic strains (Table S1). The results, illustrated in figure 1, demonstrate that the toxicity of each colicin and its capability to induce its peers are highly correlated (r = 0.84). Our results indicate that the DNA degrading colicins E2 and E7 are more potent than colicins A and K that form pores in their adversaries’ membrane, while colicins E6 and D that degrade RNA show either mild or intermediate toxicity, respectively (Figure 1). Colicin Ib, a unique pore former [7], showed intermediate toxicity levels similar to ColD.

We then tested for colicin-mediated mutual induction. To that end we used reporter-gene assays to study whether the presence of colicin extracts would result in the expression of colicins (Table 1) *E. coli* strain BZB1011. The resulting reporter strains were then exposed to colicin extracts. We employed ‘all vs. all’ combinations of colicin extracts and reporter vectors (Table 2). The results show that the presence of pore-formers (strains ColA, Ib and K), RNases (strains ColD and E6) and DNases (strains ColE2 and E7) trigger colicin expression. The prompter regions of colicins A, D, E2, E6, E7, and K are analogous, while the promoter to colicin Ib encoding operon differs [21], accordingly, the depicted expression rates measured for each colicin extract are similar, with the exception of Lum-Ib response (Table 2). Yet, the rate of toxicity and induction varies between the triggers in accord with a particular colicin’s mode of action (Figure 1 and Table 2). A significant correlation between potency and induction capacity of each colicin was observed (Figure 1), such that potent toxins, like ColE2, were strong inducers and weaker toxins, like ColA, were mild inducers. The control lysate extracted from a noncolicinogenic strain (but in every other aspect isogenic) was not toxic and induced no expression in the reporter strain (Table 2), as previously reported [18].

The highest induction rates were attributed to the most potent colicins, E2 and E7 (Figure 1). This can be explained by these colicins’ mode of action. Both cleave their target’s genomic DNA nonspecifically [7], thus inducing the DNA damage control system, namely the SOS response system, that was found to be a general regulator of all known colicins [21].

Colicin D is a tRNase that specifically cleaves the arginine anticodon loop of the tRNA isoacceptors, thus inactivating protein synthesis and leading to cell death [28]. However, the cellular response to colicin D toxification is not known. Our results showed it to be of intermediate toxicity and consequently a moderate trigger to colicin expression (Figure 1). Likewise, colicin Ib that form pores in its target membrane was moderately toxic and moderate inducer to the colicinogenic strains (Figure 1). Both colicins D and Ib may have induced the SOS response, as has been previously reported for antibiotics [29], and thus triggered colicin expression.

At the low end of the toxicity/induction scale (Figure 1), three colicins (two pore-formers and an rRNase) were mildly toxic and consequently trigger limited response from the colicinogenic strains (Figure 1). The pore-formers, colicins A and K, kill by depolarizing the cytoplasmic membrane with the formation of voltage-dependent ionic channels [30]. Although pore-formers are the most common bacterial toxins, the host-response they provoke is unknown, however, they probably do not trigger the SOS system. Similarly, the rRNA degrader colicin E6 was shown to be mildly toxic and it did not trigger colicin expression. Ribosomal RNase-type colicins provoke cell death through inactivation of the protein’s biosynthetic machinery by binding to a specific site in the ribosome [7]. Host responses to another rRNase, colicin E3, have been studied in detail, revealing a broad expression response that includes the induction of cold-shock genes, but not of SOS genes [31]. This might explain the lack of colicin expression in response to induction by colicin E6 (Figure 1).

### Colicin Competition in an Unstructured Environment

We found that colicin potency and induction highly correlate (Figure 1), which suggest that strong inducers are very toxic to their target, while weak inducers produce mild toxins. To test the competitive advantage of colicinogenic strains, we co-incubated strain ColA, tagged with a constitutive green fluorescent protein (GFP), with selected strains ranging from weak to strong inducers (ColK, ColE6, ColD and ColE7, respectively). The use of a GFP tag enabled us to follow the competition in real time.

**Figure 2:**
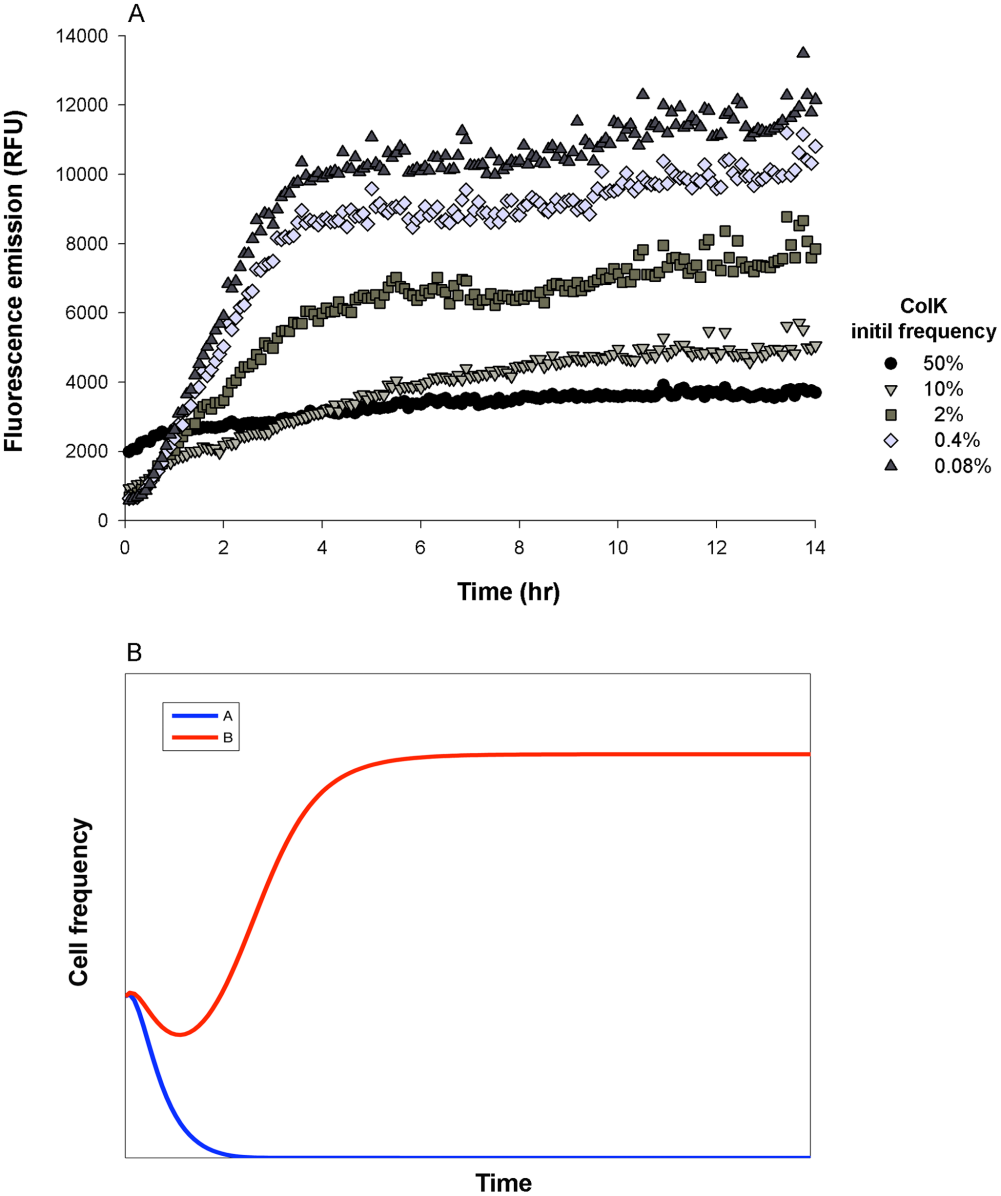
Community dynamics of bacteriocin producers in an unstractured environment. Competitions between bacteriocin producers in an unstructured environment were tested (*A*)Competition in unstrauctured habitat cwas tested empirically and (*B*) numerically. Both simulations demonstrate that competition between equal bacteriocinogenic strains (in this case mutually triggering mild bacteriocin expression) resolves in the slightly more potent bacteriocinogenic strain prevailing when initial concentrations are equal. (A) We followed the fluorescently labeled ColA over time to illustrate the competition between two pore formers (ColA and ColK) both mild inducers of colicin expression. ColK, the producer of a slightly more potent colicin, challenged ColA at various initial frequencies. At the higher starting frequencies ColK outcompeted ColA (note that ColA fluorescence did not increase over time), while at lower starting frequencies ColA outcompeted ColK. Data points are the average of three independent measurements. (B) Time evolution is illustrated by the bacteriocin producers strains A (blue line) and B (red line); both strains are mild inducers but bacteriocin B is slightly more potent. The strains were simulated to compete at equal initial frequencies and the more potent strain prevailed.

**Figure 3:**
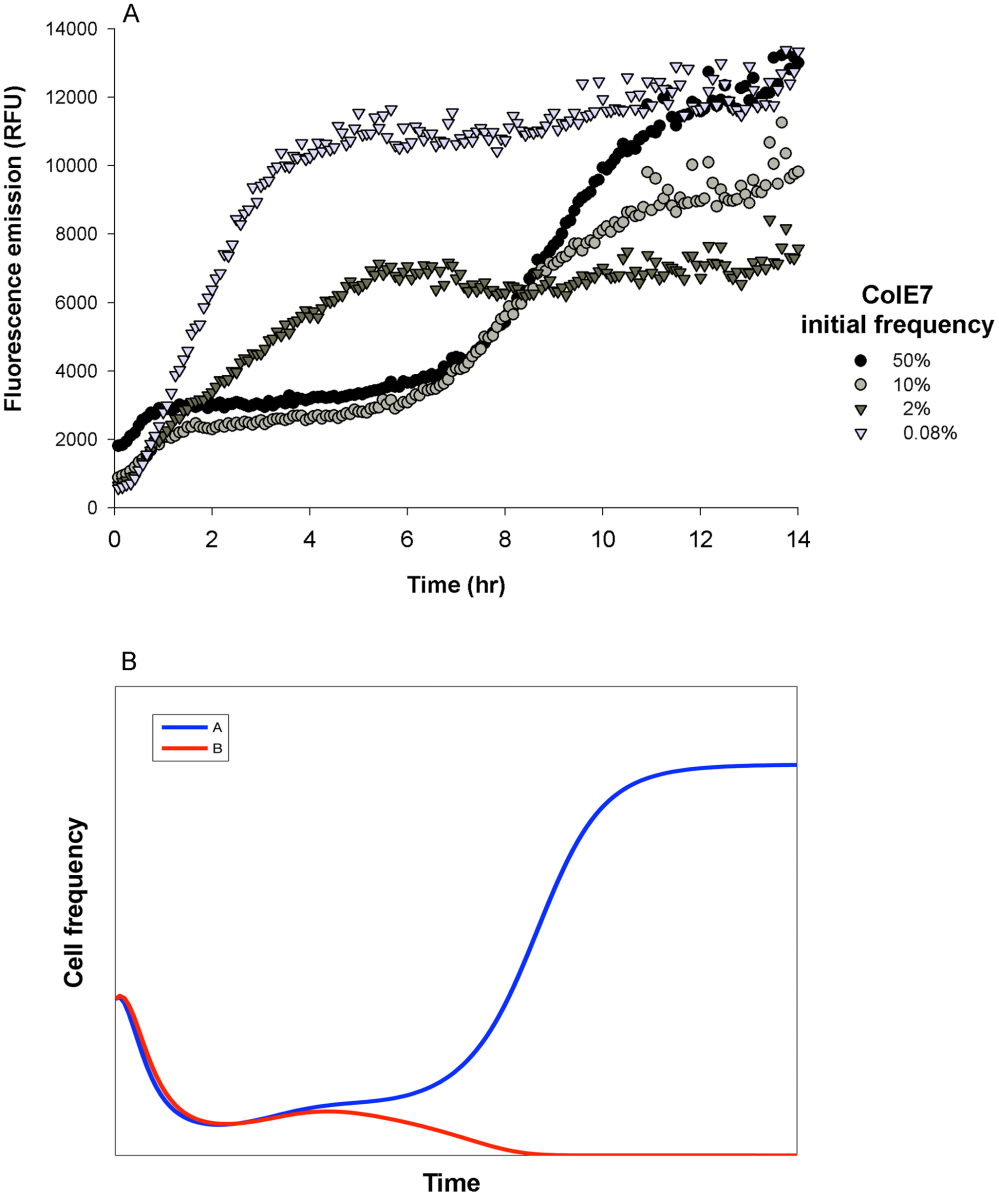
Community dynamics of bacteriocin producers in an unstractured environment. Competitions between bacteriocin producers in an unstructured environment were tested (A) empirically and (B) numerically. Both simulations demonstrate that competition between unequal bacteriocinogenic strains (one triggering mild and the other strong bacteriocin expression) resolves in the mild bacteriocinogenic strain always prevailing. (A) We followed the fluorescently labeled ColA over time to illustrate the competition between a pore former (ColA) and a DNase (ColE7) a mild and strong inducers of colicin expression, respectively. ColE7, the producer of a potent colicin, challenged ColA at various initial frequencies and was always outcompeted. At the higher starting frequencies ColA’s fluorescence was halted for a while (6 hr) but then it increased to its maximum. At lower starting frequencies, ColA outcompeted ColE7 at the onset. Data points are the average of three independent measurements. (B) Time evolution is illustrated by the bacteriocin producers strains A (blue line) and B (red line), mild and strong inducers, respectively, while strain B also produces a potent toxin. The strains were simulated to compete at equal initial frequencies and the weak inducer strain A prevailed.

The competition experiments demonstrated that the outcome of unrestricted producer-producer interactions depends on a combination of cross-induction and potency, as well as on the initial frequencies of the competing colicinogenic strains (Figures 2 and 3). When the competing strains cross-induced one another equally, either strongly [ColE2 vs. ColE7 [20]] or weakly [ColA vs. ColK or E6 (Figures 2A and S1A, respectively)], the more potent competitor eliminated its counterpart. This outcome depended on the initial frequencies of the competitors (Figures 2 and S1). Below a certain initial concentration, even potent competitors were displaced by their antagonists. This threshold concentration differed according to species: it was lower for ColK and higher for ColE6 (Figures 2A and S1A, respectively).

Next we tested the outcome of pairwise interactions between strains that differentially cross-induced one another (Figure 1). We competed strong inducers (ColE7 and D) against a weak inducer (ColA) and the respective Figures 3A and S1B show that the growth of ColA was at first halted and then resumed. We propose that upon encounter, both strains kill each other and growth fall below the detection limit; in other words, fluorescent expression is halted for over 6 h. But the competition then takes an unexpected turn, as ColA, the weak inducer, defeats it’s potent opponents, ColE7 and ColD (Figures 3A and S1B, respectively). Interestingly, the initial frequencies of the strong inducer corresponded with the final frequencies of the weak competitor, as high initial concentrations of the strong inducer resulted in the high final concentrations of the weak inducer and vice versa (Figures 3A and S1B). To explain this counter-intuitive outcome, we propose that strong induction by a potent toxin causes the weaker antagonist to increase its colicin expression, without reciprocation. The strong inducer maintains its toxin’s production at a basal level, estimated at about 3% of the cells generating colicins [9], while the weak inducers enhance their toxin production. We suspect that strain ColA responded to colicin E7’s strong induction by secreting increasing amounts of toxin. When the accumulating toxic colicin A molecules reached a certain threshold, ColA annihilated its competitor. Consequently, the strong inducer is at a disadvantage when faced with a weak opponent (Figure 3).

We used numerical simulations to reconstruct these empirical results by simulating two colicinogenic strains, A and B. Competition between the two strains resulted in stable steady states, with either strain A dominating the community and eliminating strain B, or vice versa (the density of the dominant species is 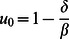). We simulated competitions between weak inducing strains, where both strains produce small quantities of their respective toxin and the toxin trigger mild expression in the competitor (Figure 2B). At equal initial frequencies, the more potent of the two strains (in this case strain B) increases in frequency and eliminates its opponent, strain A (Figure 2B). If both strains produce strong inducing toxin then the competition results in a highly toxic medium and both strains decrease in frequency (Figure S2). Subsequently, the producer of the more potent toxin (in this case strain A) recovers and eliminates its opponent, strain B, and eventually dominate the community (Figure S2).

Producer-producer dynamics bear limited resemblance to the well-studied competition between toxin-producer and toxin-sensitive strains, in which the community is bi-stable: the producer will exclude a sensitive strain if its initial concentration exceeds a certain threshold [12]. This bi-stability has been confirmed in laboratory experiments, where under well-mixed conditions, changing the initial density of the producer displaces its sensitive competitor only when above a critical frequency [12]. But what happens when one inducer is weak while the other is strong? In Figure 3B, strain B (*u_B_*) is a strong inducer to strain A (*u_A_*) toxin expression. A pairwise competition, with both antagonists starting at equal concentrations, initially results in a drastic decrease in the concentrations of both strains as the toxic medium prevents their growth. But as A is able to produce more toxin (*c_A_*) in response to strong induction by strain B’s toxin (*c_B_*) it become very toxic, eliminates B and eventually dominates the community.

**Figure 4:**
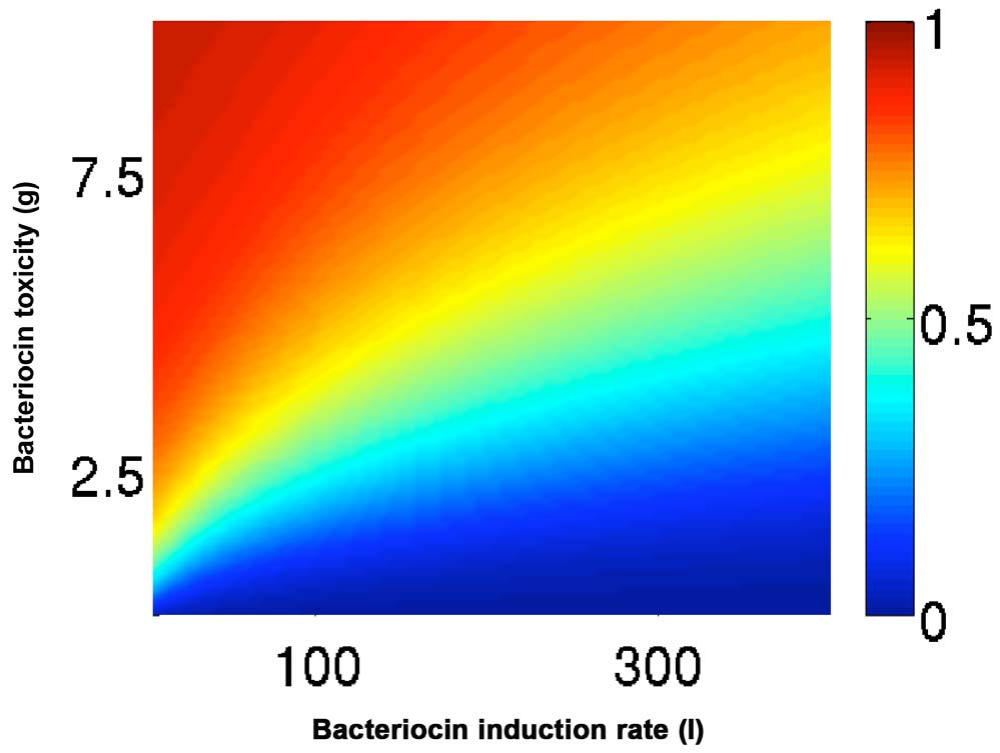
Frequency-dependent community dynamics of bacteriocin producers in an unstructured environment. We initialized the system such that the sum of the densities was constant, 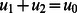, and the initial relative frequency varied 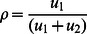
_._ We observed a critical frequency, ρc, above which *u_1_* eventually dominates. ρc was plotted for a range of toxicity (γ_A_) and induction ability (*l*_A_) values. As expected, ρc increased with both γ_A_ and *l*_A_. Parameters: γ_B_ = 2, *l*_A_ = 40.

We then investigated the influence of the variables on the fate of the system by examining the role of the induction strength of a colicin produced by strain A, *l_A_*, and of the potency of strain A’s toxin, γ_A_, while keeping *l_B_* and γ_B_ constant (Table 3). For all the tested variables, both strains may dominate, but the initial conditions that lead to this outcome (the basin of attraction) may vary (Figure 4). In particular, if we initialize the system such that 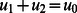, and vary the initial relative density 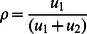, then a critical relative density, ρc, is realized, above which *u_A_* eventually dominates. Figure 4 demonstrates ρc for various values of *l_A_* and *γ_A_*; as expected, the value of ρc increases with both *γ_A_* and *l_A_*.

### Colicin Competition in a Structured Environment

In a spatially structured environment, some regions in space may be dominated by one species, while its competitor dominates adjacent regions. This state is transient. We suggest that the fronts at the interface between these two regions move until only one species is left. But in contrast to well-mixed environments, structured environments where interactions are local favor lengthy resolution and the species coexist for extended periods [4], [19].

**Figure 5:**
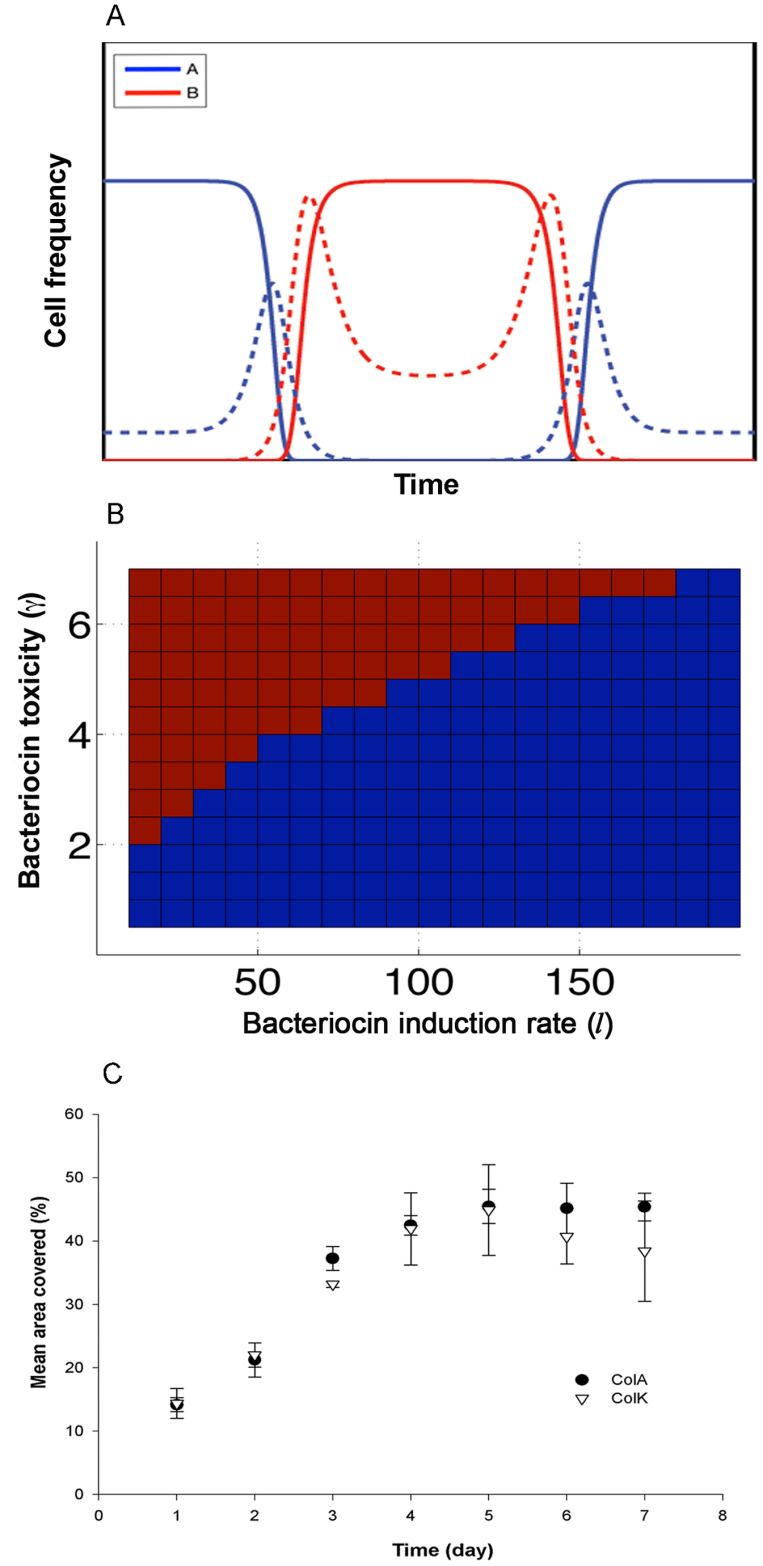
Community dynamics of bacteriocin producers in a structured environment. Competitions between bacteriocin producers in a structured environment were tested (A, B) numerically and (C) empirically. (A) Snapshot of local competition between two bacteriocinogenic strains. One, *u_A_*, dominates in some regions in space, while its competitor, *u_B_*, dominates in others. These regions are separated by fronts that are moving further away from the dominant strain, *u_B_*. Bacteriocin concentrations are higher near the fronts due to mutual induction. In (B), the winner of the pairwise competition was plotted for various values of the test strain induction, *l*_A_, and sensitivity, γ_1_. Blue indicates domination of strain A, *u_A_*, and red indicates domination of its’ competitor *u_B_*. (C) A static plate environment was initiated by randomly depositing 24 droplets from pure cultures of the colcinogenic strains ColA and ColK. The changing spatial pattern of the community was documented and the mean area of each strain’s coverage of the plate surface calculated. The aerial coverage of the strains was shown to remain invariable over time. Data points are average of two independent measurements and the bars represent the deviation from the average.

Spatial interactions of bacteriocin-producer and -sensitive strains have been described both empirically and theoretically [4], [12]. In spatially structured environment this intransitive system, the producing strain, can always defeat the sensitive strain, regardless of its initial frequency provided that the interactions and dispersal occur on a local scale. Producer-producer interactions present a similar outcome. Our theoretical model suggests that some regions in space may be dominated by strain A (*u_A_*), others by strain B (*u_B_*). As already noted, this state is transient as the fronts at the interface between regions (“no man’s land”) move until only one species is left (Figure 5A and S4). In one dimension (1D), the fronts always retreats with the advance of the dominant species, hence, the dominant species is independent of the initial frequency, provided that there is at least one region in space where they are able to dominate.

In two dimensions (2D), the interactions present a more complex picture, as the curvature of both fronts may affect the dynamics. Specifically, the bacteria in the boundaries of the convex regions are exposed to more bacteriocin, and therefore are at a selective disadvantage. However, if the space the bacteria occupy is wide enough, the curved fronts might eventually flatten and the final outcome will mirror the 1D system.

Figure 5B demonstrates bacteriocin producing species B that is induced by a wide variety of induction strengths (changing *l_A_*) and potencies (γ_A_) of a putative bacteriocin marked as A. As predicted, the putative A strain produces bacteriocin A and dominates at higher values of *l_A_* and lower values of γ_A_. The speed at which the dominant species eventually fixates is slower in 2D as the initial curvature may lead to exclusion of the dominant species in some regions in space, while recovery takes place only later, as the fronts straighten.

Empirical tests done when the strains were locally interacting in spatially structured environments showed no resolution, with the species coexisting during the experiments (Figures 5C and S3). When Colicin A was competed against Colicins K, E6, or ColE7 (Table 1), the strains coexisted, maintaining “territorial integrity” throughout the experiment. This suggests that interactions between bacteriocin producers can result in temporary coexistence. Similarly, bacteriocin-mediated interactions tested in co-caged mice, each carrying a different colicinogenic strain in its colon, did not resolve for over 4 months [20], whereas sensitive, resistant, and producer strains were competitively displaced in a similar murine model [14].

## Discussion

In the last decade, there has been a surge in the estimates of factors that affect biodiversity [32], [33], [34]. It has been suggested that in microbial communities, antibiotic-mediated interactions play a key role in maintaining biodiversity, possibly via the existence of intransitive fitness interactions and via the spatial structure within habitats [33], [34]. In addition it has been suggested that antibiotics present the producer cells with both a weapon and a liability. They are, simultaneously, lethal to targets cells and impose an energetic cost on the producers who carry the genes encoding antibiotic production [6], [34]. We hypothesized that an additional factor, antibiotic-mediated induction, is at play, changing the outcome in producer-producer interactions. We suggest that competition between producers would be determined by the potency and strength of the inducing antibiotic in a frequency-dependent manner, as has been previously reported [20], yet the resolution will not necessarily be to the benefit of the potent strain.

Bacteriocins are meant to harm cells that are closely related to the producer and compete the producer over the same resources. However, when producers are interacting, the bacteriocin of one may not only harm the other, but may also serve to trigger its bacteriocin production, arming the competitor for counter-attack. We have found that bacteriocins cross-induce each other’s expression in a manner that is positively correlated with their toxicity; in other words, the potency of a bacteriocin and its induction ability go hand in hand (Figure 1). In a pairwise competition of evenly matched antagonists (exercising either weak or strong induction), the more toxic competitor will prevail provided it is initially frequent (Figures 2 and S2). This frequency-dependent outcome is consistent with reports of unrestricted interactions between bacteriocin -producer and -sensitive strains [12], [13], [15], [35], [36]. There is, however, one significant difference – we attribute the outcome in the case of producer-sensitive strains interplay to the high energetic cost incurred by the producer strains [9], [12], [35], while in the producer-producer interplay both strains carry a similar cost (Table 2). Thus, the outcome merely reflects differences in colicin lethality.

We further explored another scenario in which the competition is not equal, for instance, when a weak inducer faces a stronger bacteriocin producer. Contrary to our prediction, the weaker opponent displaced the strong inducer in a frequency-dependent manner (Figure 3). This seemingly counter-intuitive result might be explained by the significant role of antibiotic induction in competitive interactions. When strains that produce a strong inducing bacteriocin are fairly common, they trigger their antagonists to produce much more bacteriocin without reciprocation, i.e., the weak producer mildly triggers its opponent to produce bacteriocin. Therefore, the net production of the producers of weakly inducing bacteriocin is considerably increased, bringing about the defeat of their strong inducing antagonists which, for lack of external induction, do not alter their basal bacteriocin production rate [9].

The model described here portrays a unique interplay between bacteriocin producers and predicts selection toward weak inducers (Figure 4). A survey of known bacteriocins [37], and in particular of colicin [300, reveals that this strategy was implemented by most bacteriocins. Over 70% of the colicins dominating natural populations are weak inducers; 13 out of the 22 colicins identified to date, are pore-formers [7], [30], the mode of killing, which we found to mildly trigger colicin expression (Figure 1 and Table 2). Three additional colicins degrade the target cells’ ribosomal RNA [7] and are thus suspected of being mild inducers, similar to ColE6 (Figure 1 and Table 2). This leaves only six colicins as potential strong inducers of their competitors, namely, the four DNase colicins (following the example of ColE2 and E7) and the two tRNase colicins (following ColD), affirming the selection toward weak inducers. Interestingly, all characterized bacteriocins produced by Gram-positive bacteria (such as nisin, mutacin or pediocin) form pores in the membranes of their targets [8,367, indicating strong selection for this trait. However, it remains to be seen what response these bacteriocin-producing strains induce in their competitors.

To further test our model, we simulated induction-mediated interactions in a structured environment. It has been suggested that spatial structure factor in shaping biodiversity [4], [11], [14], and this idea was supported by our data. We found that local interactions resolve very slowly (Figure 5), as competition between species occurs only in the colony frontiers, i.e., “no man’s land” between the competitors’ territories. Moreover, the outcome of local interplay was not frequency-dependent (Figure 5B). Therefore, competition in any structured environment, though following the same rules of induction and toxicity dominance, results in much slower dynamics and prolonged coexistence, i.e., both populations persist over extended periods. In natural environments, such as the mammalian gut, this extended persistence might allow for the introduction of additional players to the community by migration or evolution, thus enhancing microbial diversity.

## Supporting Information

Figure S1 **Community dynamics of colicin producers in an unstractured environment.** Competitions between bacteriocin producers in an unstructured environment were tested to illustrate the density-dependent competition between various strains’ inducers. (*A*) We followed the fluorescently labeled ColA over time to illustrate the competition between a pore former and an rRNase (ColA and ColE6, respectively) both mild inducers of colicin expression. ColE6, the producer of a slightly more potent colicin, challenged ColA at various initial frequencies. At most starting frequencies ColE6 outcompeted ColA (note that ColA fluorescence did not increase over time). Only at very low starting frequency ColA outcompeted ColK. (*B*) We competed the fluorescently labeled pore former (ColA) and a tRNase (ColD) a mild and intermediate inducers of colicin expression, respectively. ColD, the producer of a potent colicin, challenged ColA at various initial frequencies and was always outcompeted. At the higher starting frequencies ColA’s fluorescence was halted for a while but then it increased to its maximum. At lower starting frequencies, ColA outcompeted ColD at the onset. (*C*) We competed the fluorescently labeled pore former (ColA) and a colicin-free isogenic strain, used as a control. At all initial concentrations used ColA outcompetes the colicin-free strain. Data points are the average of three independent measurements.
(DOCX)

Figure S2 **Community dynamics of bacteriocin producers in an unstractured environment.** Time evolution is illustrated by the bacteriocin producers strains A (blue line) and B (red line); both strains are strong inducers but bacteriocin B is slightly more potent. The strains were simulated to compete at equal initial frequencies and the more potent strain prevailed.
(DOCX)

Figure S3 **Community dynamics of bacteriocin producers in a structured environment.** Static-plate environment was initiated by randomly depositing 24 droplets from pure culture of strains ColA and ColE6 (*A*). A separate set of experiments explored the interactions between ColA and ColE7 (*B*). The changing spatial pattern of the community was documented and the mean area of each strain’s coverage of the plate surface calculated. The aerial coverage of the strains was shown to remain invariable throughout the experiment. Data points are average of two independent measurements and the bars represent the deviation from the average.
(DOCX)

Figure S4 **Time evolution of two competing species in a structured environment.** Fronts separate regions dominated by different species. These fronts are moving away from the dominant species (A), until its competitor is almost eliminated (B).
(DOCX)

Table S1 **Growth rate of** ***E. coli*** **strains.** Data presented as mean ± standard deviation (rounded to two decimal points).
(DOCX)

Table S2 **Primers used in this study.**
(DOCX)

Table S3 **Colicin reporter vector induction by 0.5 ng mL^−1^ mitomycin C.** Data presented as mean ± standard deviation.
(DOCX)
