# Supplementary material for: The Weak Shall Inherit: Bacteriocin-Mediated Interactions in Bacterial Populations
Source: PLoS One. 2013 May 21;8(5):e63837. doi: 10.1371/journal.pone.0063837 (PMC3660564; doi:10.1371/journal.pone.0063837)
Supplement: Figure S1 — Community dynamics of colicin producers in an unstractured environment. Competitions between bacteriocin producers in an unstructured environment were tested to illustrate the density-dependent competition between various strains’ inducers. (A) We followed the fluorescently labeled ColA over time to illustrate the competition between a pore former and an rRNase (ColA and ColE6, respectively) both mild inducers of colicin expression. ColE6, the producer of a slightly more potent colicin, challenged ColA at various initial frequencies. At most starting frequencies ColE6 outcompeted ColA (note that ColA fluorescence did not increase over time). Only at very low starting frequency ColA outcompeted ColK. (B) We competed the fluorescently labeled pore former (ColA) and a tRNase (ColD) a mild and intermediate inducers of colicin expression, respectively. ColD, the producer of a potent colicin, challenged ColA at various initial frequencies and was always outcompeted. At the higher starting frequencies ColA’s fluorescence was halted for a while but then it increased to its maximum. At lower starting frequencies, ColA outcompeted ColD at the onset. (C) We competed the fluorescently labeled pore former (ColA) and a colicin-free isogenic strain, used as a control. At all initial concentrations used ColA outcompetes the colicin-free strain. Data points are the average of three independent measurements. (DOCX) [file pone.0063837.s001.docx]

**Figure S1.**


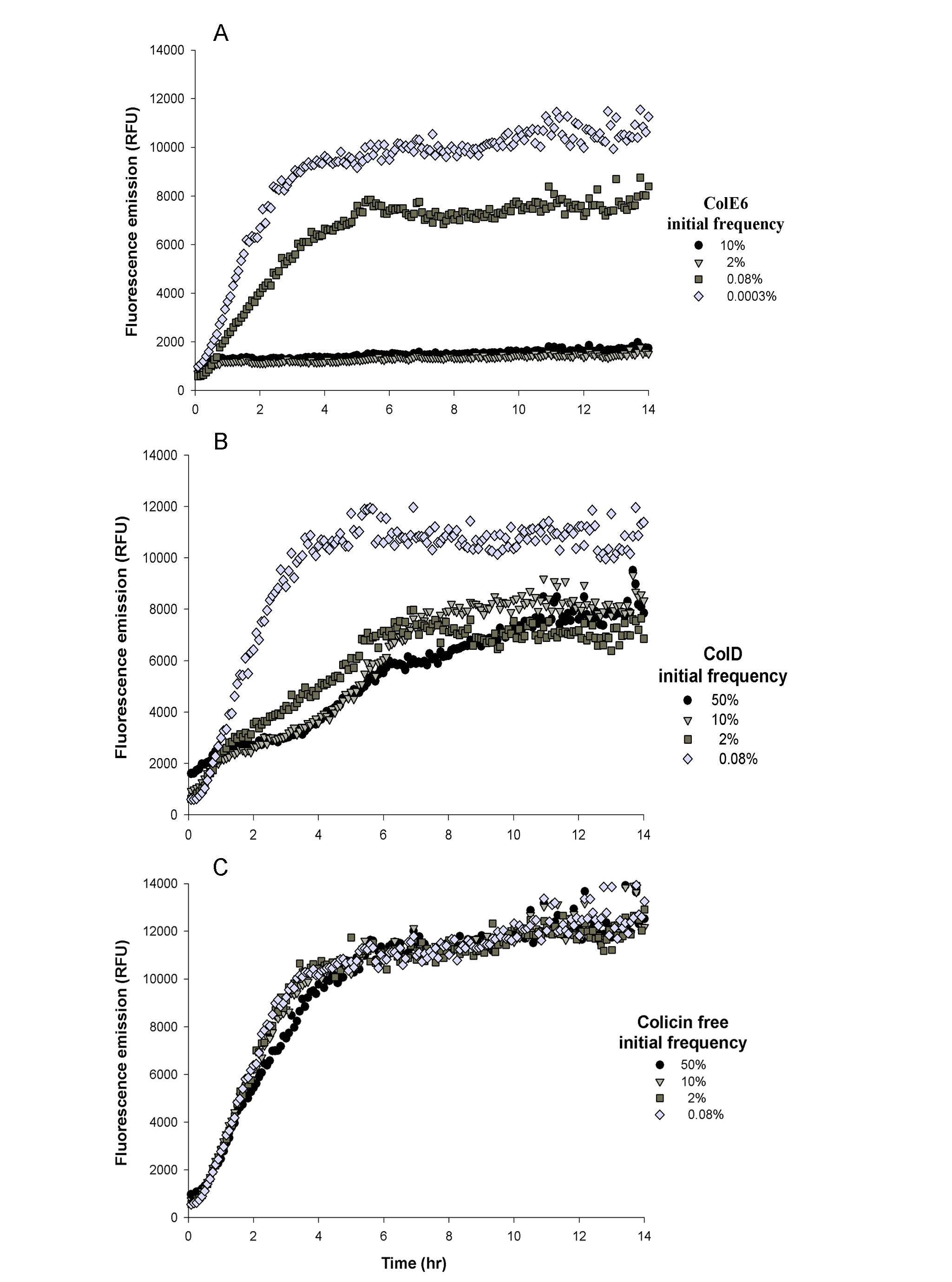


**Figure S1.** **Community dynamics of colicin producers in an unstractured environment.** Competitions between bacteriocin producers in an unstructured environment were tested to illustrate the density-dependent competition between various strains' inducers. (*A*) We followed the fluorescently labeled ColA over time to illustrate the competition between a pore former and an rRNase (ColA and ColE6, respectively) both mild inducers of colicin expression. ColE6, the producer of a slightly more potent colicin, challenged ColA at various initial frequencies. At most starting frequencies ColE6 outcompeted ColA (note that ColA fluorescence did not increase over time). Only at very low starting frequency ColA outcompeted ColK. (*B*) We competed the fluorescently labeled pore former (ColA) and a tRNase (ColD) a mild and intermediate inducers of colicin expression, respectively. ColD, the producer of a potent colicin, challenged ColA at various initial frequencies and was always outcompeted. At the higher starting frequencies ColA’s fluorescence was halted for a while but then it increased to its maximum. At lower starting frequencies, ColA outcompeted ColD at the onset. (*C*) We competed the fluorescently labeled pore former (ColA) and a colicin-free isogenic strain, used as a control. At all initial concentrations used ColA outcompetes the colicin-free strain. Data points are the average of three independent measurements.
