# Supplementary material for: The Weak Shall Inherit: Bacteriocin-Mediated Interactions in Bacterial Populations
Source: PLoS One. 2013 May 21;8(5):e63837. doi: 10.1371/journal.pone.0063837 (PMC3660564; doi:10.1371/journal.pone.0063837)
Supplement: Figure S2 — Community dynamics of bacteriocin producers in an unstractured environment. Time evolution is illustrated by the bacteriocin producers strains A (blue line) and B (red line); both strains are strong inducers but bacteriocin B is slightly more potent. The strains were simulated to compete at equal initial frequencies and the more potent strain prevailed. (DOCX) [file pone.0063837.s002.docx]

**Figure S2.**


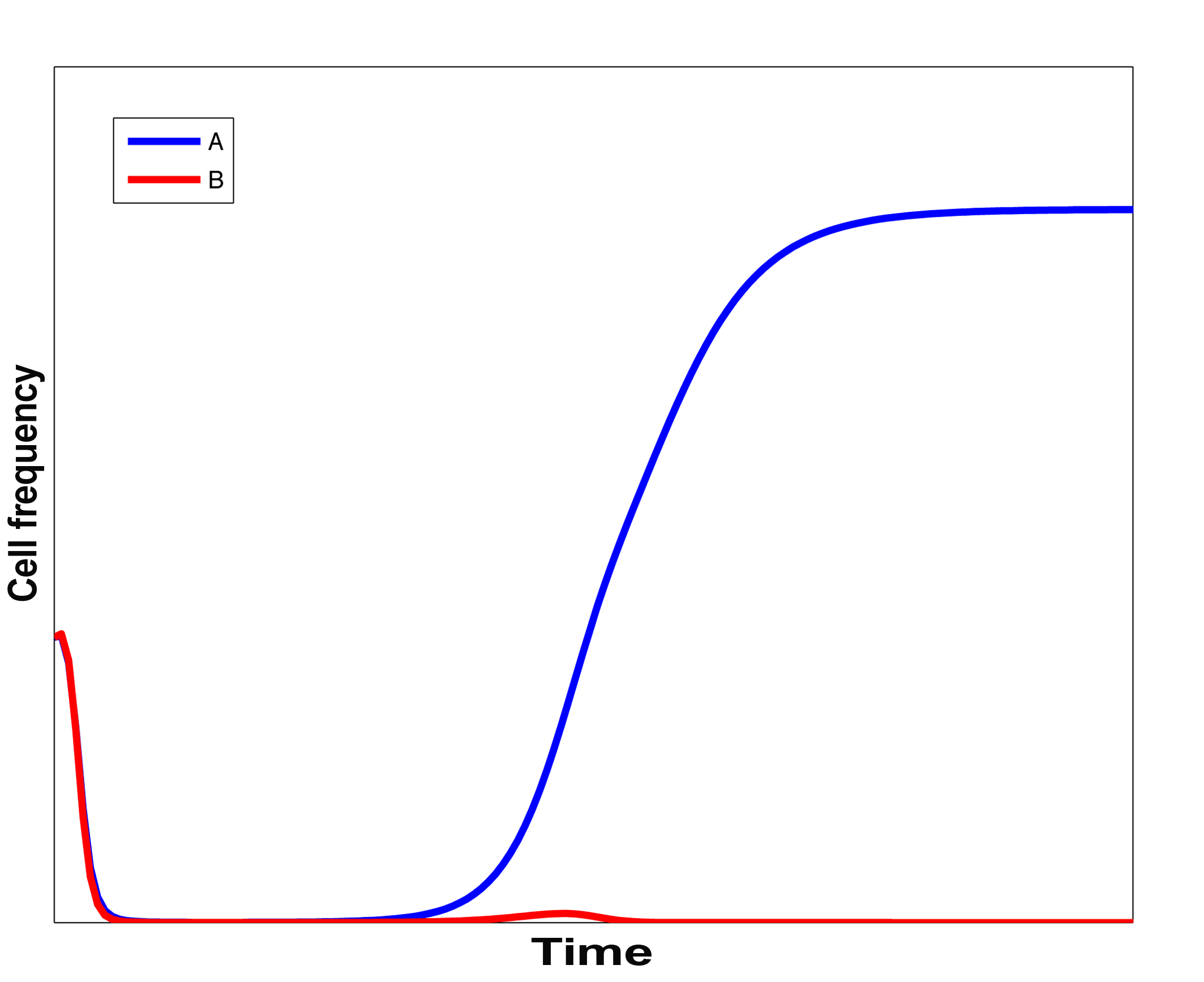


**Figure S2.** **Community dynamics of bacteriocin producers in an unstractured environment.** Time evolution is illustrated by the bacteriocin producers strains A (blue line) and B (red line); both strains are strong inducers but bacteriocin B is slightly more potent. The strains were simulated to compete at equal initial frequencies and the more potent strain prevailed.
