# Supplementary material for: The Weak Shall Inherit: Bacteriocin-Mediated Interactions in Bacterial Populations
Source: PLoS One. 2013 May 21;8(5):e63837. doi: 10.1371/journal.pone.0063837 (PMC3660564; doi:10.1371/journal.pone.0063837)
Supplement: Figure S3 — Community dynamics of bacteriocin producers in a structured environment. Static-plate environment was initiated by randomly depositing 24 droplets from pure culture of strains ColA and ColE6 (A). A separate set of experiments explored the interactions between ColA and ColE7 (B). The changing spatial pattern of the community was documented and the mean area of each strain’s coverage of the plate surface calculated. The aerial coverage of the strains was shown to remain invariable throughout the experiment. Data points are average of two independent measurements and the bars represent the deviation from the average. (DOCX) [file pone.0063837.s003.docx]

**Figure S3.**


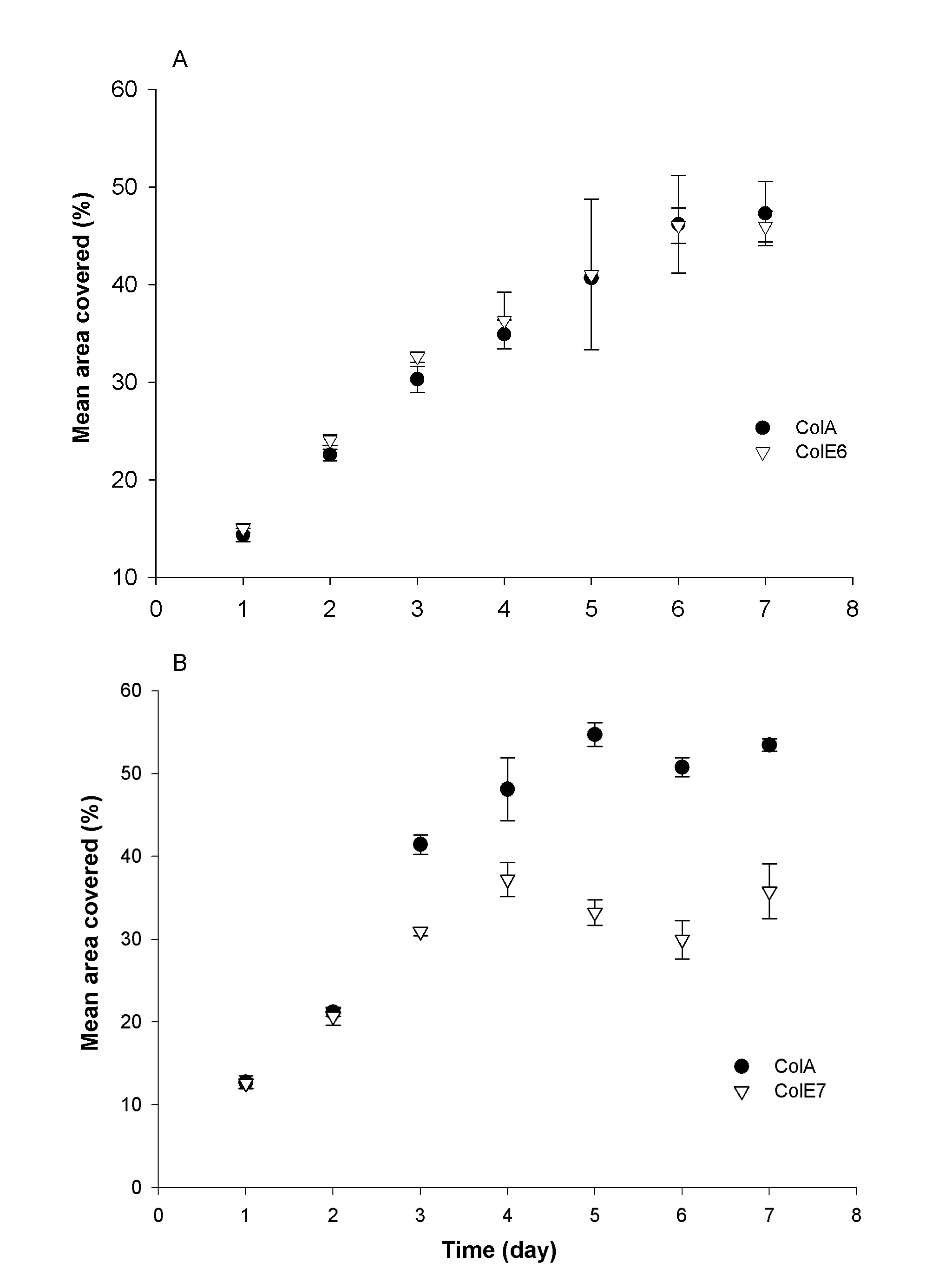


**Figure S3.** **Community dynamics of bacteriocin producers in a structured environment.** Static-plate environment was initiated by randomly depositing 24 droplets from pure culture of strains ColA and ColE6 (*A*). A separate set of experiments explored the interactions between ColA and ColE7 (*B*). The changing spatial pattern of the community was documented and the mean area of each strain's coverage of the plate surface calculated. The aerial coverage of the strains was shown to remain invariable throughout the experiment. Data points are average of two independent measurements and the bars represent the deviation from the average.
