# Supplementary material for: The Weak Shall Inherit: Bacteriocin-Mediated Interactions in Bacterial Populations
Source: PLoS One. 2013 May 21;8(5):e63837. doi: 10.1371/journal.pone.0063837 (PMC3660564; doi:10.1371/journal.pone.0063837)
Supplement: Figure S4 — Time evolution of two competing species in a structured environment. Fronts separate regions dominated by different species. These fronts are moving away from the dominant species (A), until its competitor is almost eliminated (B). (DOCX) [file pone.0063837.s004.docx]

**Figure S4.**


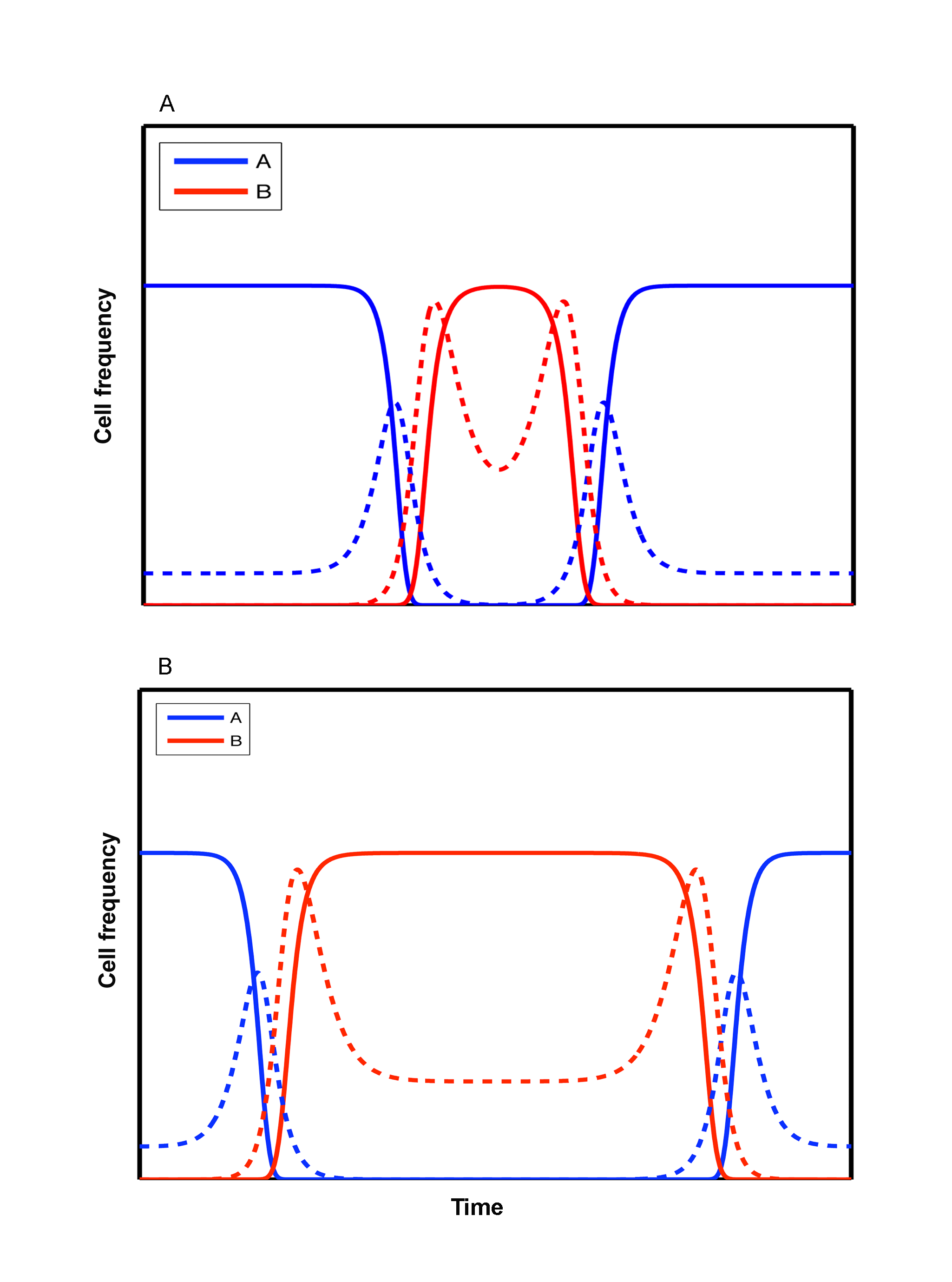


**Figure S4.** **Time evolution of two competing species in a structured environment.** Fronts separate regions dominated by different species. These fronts are moving away from the dominant species (A), until its competitor is almost eliminated (B).
