# Supplementary material for: The Weak Shall Inherit: Bacteriocin-Mediated Interactions in Bacterial Populations
Source: PLoS One. 2013 May 21;8(5):e63837. doi: 10.1371/journal.pone.0063837 (PMC3660564; doi:10.1371/journal.pone.0063837)
Supplement: Table S1 — Growth rate of E. coli strains. Data presented as mean ± standard deviation (rounded to two decimal points). (DOCX) [file pone.0063837.s005.docx]

**Table S1**. Growth rate of *E. coli* strains. Data presented as mean ± standard deviation (rounded to two decimal points)

| ***E. coli* strains** | **Growth rate μ^*^** |
| --- | --- |
| ColD | 0.22 ± 0.08 |
| ColE6 | 0.25 ± 0.06 |
| ColE7 | 0.20 ± 0.05 |
| ColK | 0.24 ± 0.05 |
| pBR-ColA::pUA*rrnB* | 0.22 ± 0.003 |
| Plasmid-free | 0.26 ± 0.007 |

^*^Growth rate is expressed in generations per hour.
