# Supplementary material for: The Weak Shall Inherit: Bacteriocin-Mediated Interactions in Bacterial Populations
Source: PLoS One. 2013 May 21;8(5):e63837. doi: 10.1371/journal.pone.0063837 (PMC3660564; doi:10.1371/journal.pone.0063837)
Supplement: Table S3 — Colicin reporter vector induction by 0.5 ng mL−1 mitomycin C. Data presented as mean ± standard deviation. (DOCX) [file pone.0063837.s007.docx]

**Table S3**. Colicin reporter vector induction by 0.5 ng mL^-1^ mitomycin C. Data presented as mean ± standard deviation.

| ***E. coli* strains** | **Mean light emission** |
| --- | --- |
| pDEW201 | 1.74 ± 0.13 |
| Lum-A | 12.44 ± 5.39 |
| Lum-D | 51.51 ± 10.39 |
| Lum-E2 | 47.92 ± 2.95 |
| Lum-E6 | 48.83 ± 8.26 |
| Lum-E7 | 47.25 ± 5.13 |
| Lum-Ib | 8.26 ± 1.62 |
| Lum-K | 47.62 ± 2.28 |
